# Supplementary material for: Myocardin-Related Transcription Factor Mediates Epithelial Fibrogenesis in Polycystic Kidney Disease
Source: Cells. 2024 Jun 5;13(11):984. doi: 10.3390/cells13110984 (PMC11172104; doi:10.3390/cells13110984)
Supplement: Supplementary file 1 [file cells-13-00984-s001.zip › cells-2996033-supplementary.pdf]

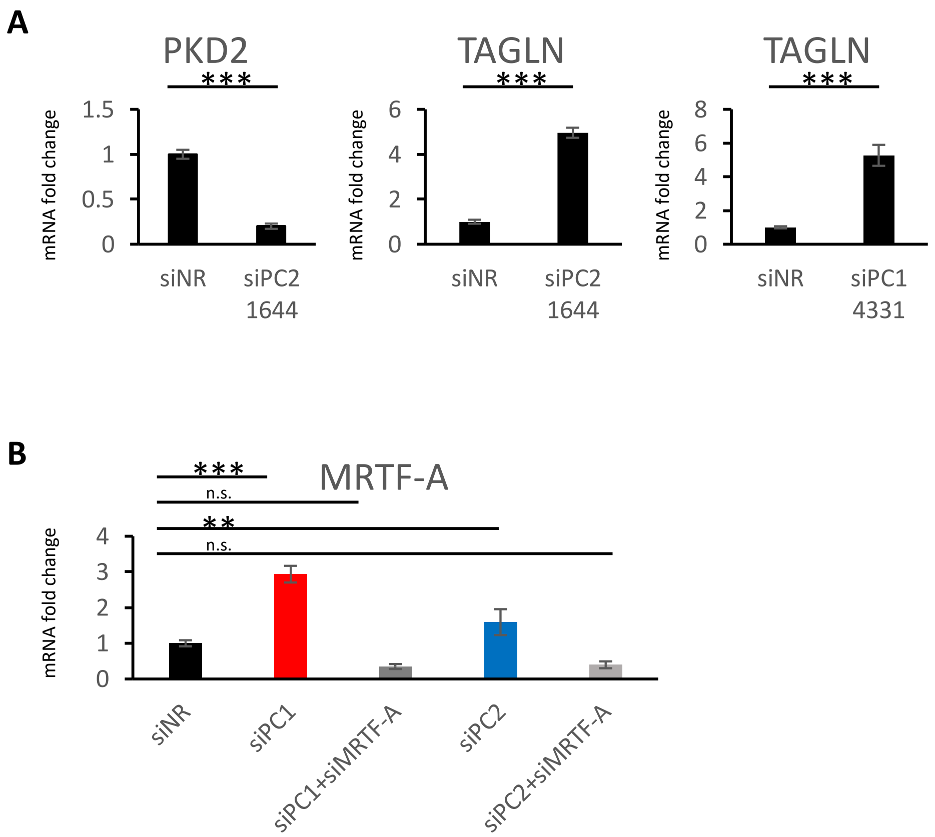

Supplementary Figure S1. Experimental setup. (**A**) siPC1-1433 and siPC2-1644 are a second set of *PC1*- and *PC2*-targeting siRNAs (respectively). Transfection of either siPC1-1433 (150 nM) or PC2-1644 (100 nM) enhanced *TAGLN* expression. qPCR data are shown. (**B**) *MRTF-A* mRNA was quantified by RT-qPCR. siPC1 or siPC2 knockdown did not alter the efficiency of MRTF-A silencing (n = 3, n.s. non-significant, ** $p < 0.01$, ***$p<0.001$).

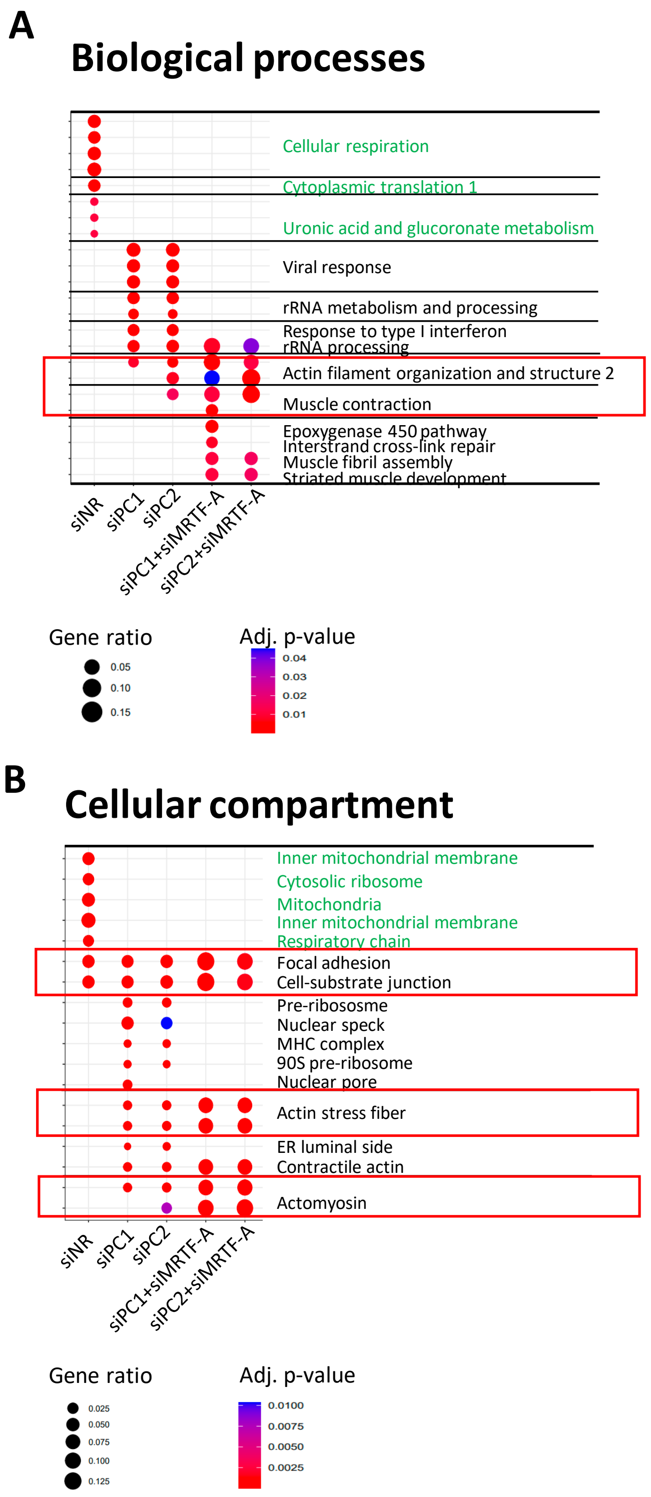

Supplementary Figure S2. PKD-linked cytoskeletal changes are regulated by MRTF. Differentially expressed genes were determined as detailed in Supplementary Methods, and were subjected to enrichment analysis of Biological Processes (**A**) and Cellular Compartment (**B**), using ClusterProfiler in R. Categories that were depleted in siPC1 and siPC2 conditions (compared to siNR) are shown in green. Further, selected top categories that were enriched in siPC1 or siPC2 conditions (compared to siNR) are listed in the siPC1 and siPC2 columns. Significant decreases compared to these in siMRTF-treated samples are shown in the next two columns. Note that biological processes that overlap between PC knockdown conditions and the siPC+siMRTF-A conditions were related to cytoskeleton organization and cell contractility (red rectangles).

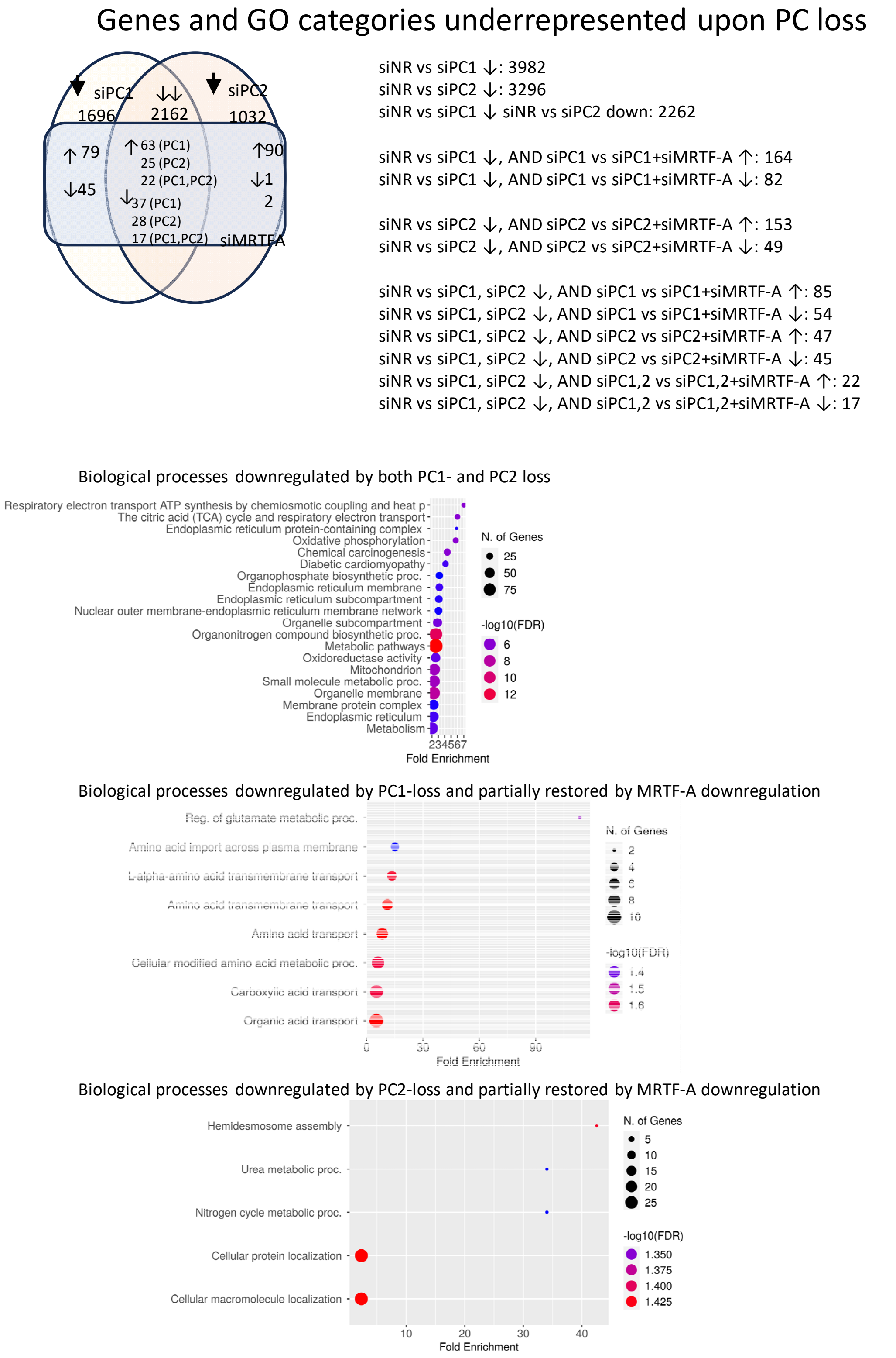

Supplementary Figure S3. PC1 and PC2 loss-specific underrepresented biological processes and their MRTF-dependence**.** Top panel: Epithelial cells knocked down for PC1 and PC2 transcriptionally underrepresented several metabolic pathways. Lower panels: Out of these, MRTF-A silencing partially mitigates amino acid transport across plasma membrane (siPC1).

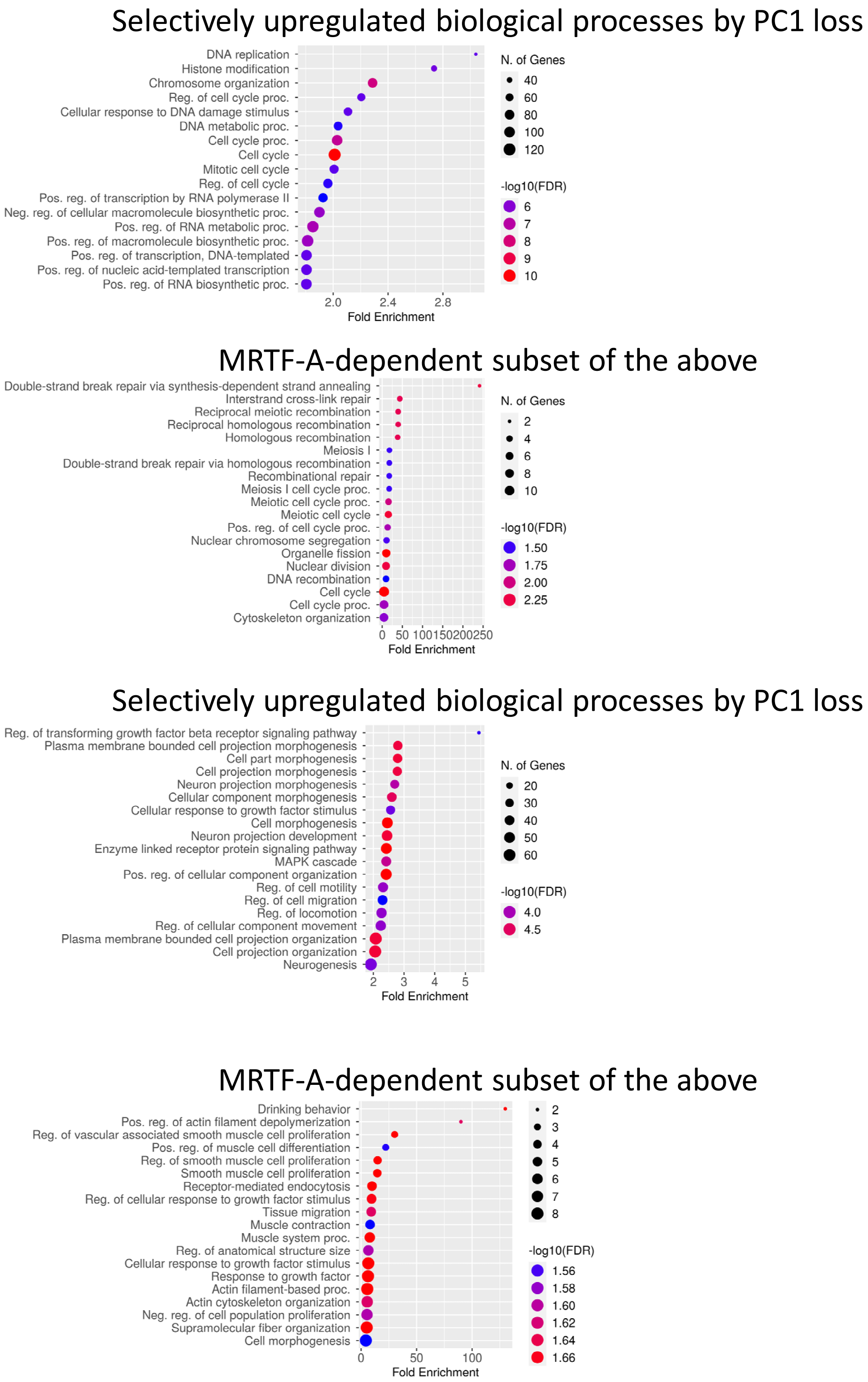

Supplementary Figure S4. PC1 and PC2 loss-specific biological processes. DEG searched for transcripts that were upregulated upon PC1 loss but were unaffected by PC2 loss, or vice versa. For PC1, loss-related biological processes centered around DNA replication and transcription (top panel). Within this transcript set, MRTF-dependent genes were grouped in DNA recombination-related repair. Interestingly, PC2-specific upregulated categories remained to be linked to cell projection/cytoskeletal reorganization/cell motility. The MRTF-dependent subset is predicted to regulate the actin cytoskeleton, cell contractility, and muscle development.

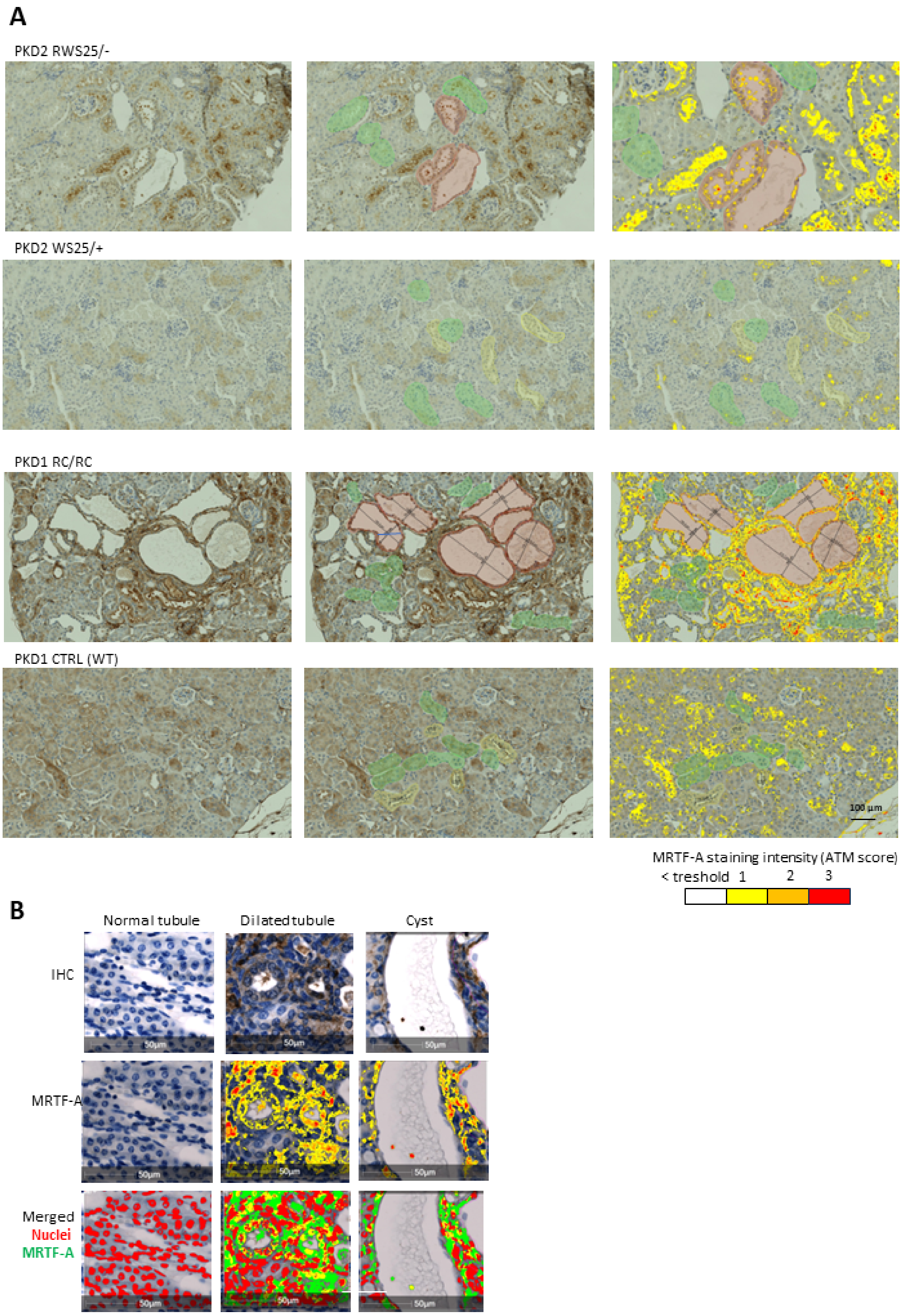

Supplementary Figure S5. Expression analysis methods. (**A**) ATM within the Area Quantification Module measured MRTF-A/DAB signal and colored each pixel according to its DAB optical density. Yellow to red color gradient corresponds to increasing MRTF-A expression, as indicated. Green and yellow masks show selected normal tubules without or with lumen, respectively. Red masks depict visibly enlarged tubules; lumen measurements are given in µm. Scale bar corresponds to all panels. (**B**) Automated Area Quantification Module was trained to recognize the hematoxylin-stained nuclei and to depict MRTF-A expression, as in (**A**, middle row panels). The bottom panels show a superimposed image of the nuclear mask and the MRTF-A-specific stain. Here red indicates nuclei, green indicates MRTF-A and yellow depicts nuclear MRTF-A.

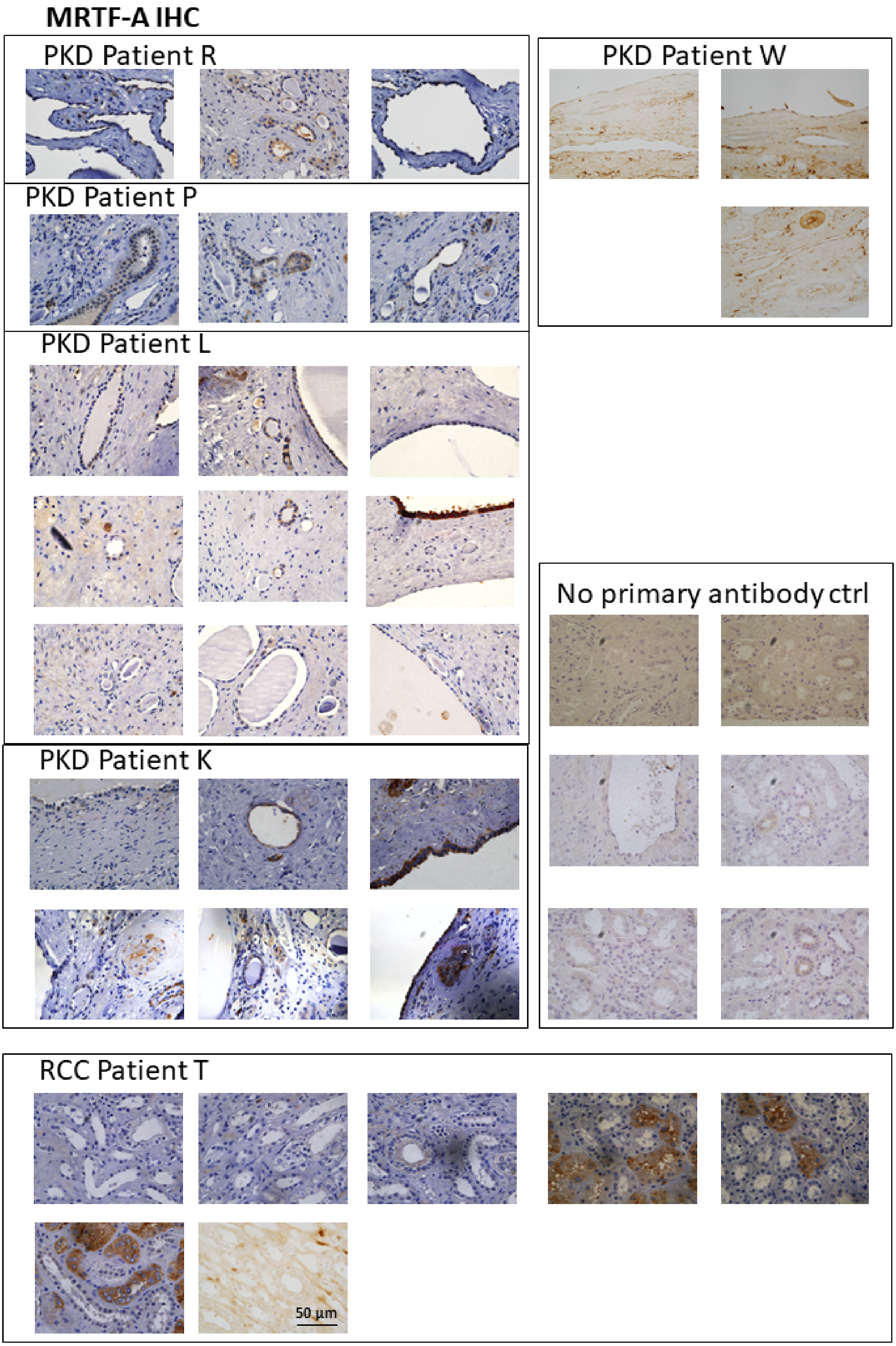

MRTF-A IHC
PKD Patient R
PKD Patient P
PKD Patient L
PKD Patient K
PKD Patient W
No primary antibody ctrl
RCC Patient T
50 µm

Supplementary Figure S6. ADPKD patients' cystic epithelium shows enhanced MRTF-A expression**.** Five ADPKD patients (patient R, P, L, K, and W) and control (normal kidney tissue from a clear cell renal cell carcinoma patient) specimens were assessed for MRTF-A expression by IHC. Nuclear expression of MRTF-A was observed in all ADPKD patients, albeit the extent varied. The cystic epithelial wall showed enhanced MRTF expression compared to the normal tubules within the same patient's specimens and to the normal tubules of the RCC patient. Scale bar corresponds to all panels.

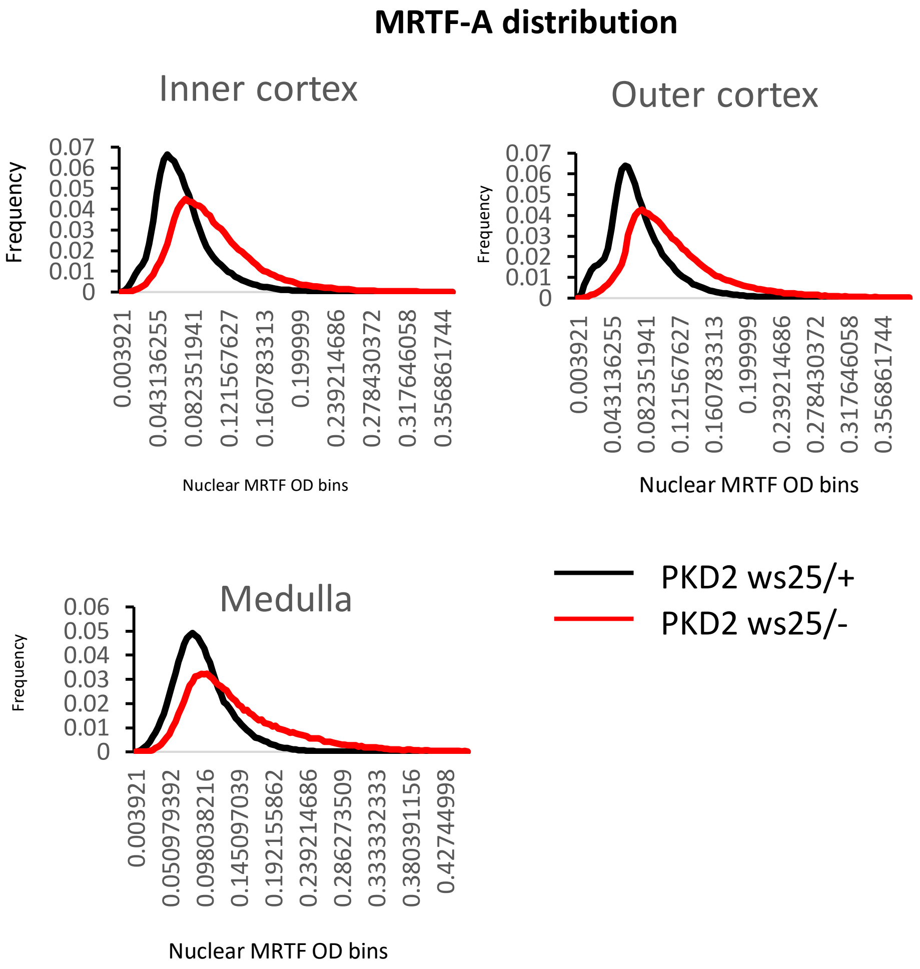

Supplementary Figure S7. Distribution curves show the frequency of nuclei with increasing MRTF-A expression**.** The inner cortex, outer cortex, and medulla were assessed separately in *Pkd2* WS25/− (3 months old, n = 5) and control P*kd2* WS25/+ (3 months old, n = 5) animals.
